# Modern Methods of Treatment

**Published:** 1912-03-23

**Authors:** 


### MODERN METHODS OF TREATMENT.

### How they Affect the Hospitals and their Patients.

It is a well-known fact that the upkeep of the modern hospital is getting more costly every year. Various causes contribute towards this increased necessary expenditure, some of them well known to the public by reason of the outstanding importance of the facts, others less well known because only those intimately associated with institutional work are in a position adequately to appreciate them. In a series of articles on "The Making of the Modern Hospital" we have endeavoured to detail some of the institutional causes which account for this increased cost of upkeep. There are also subsidiary questions which influence the institutional budget owing to the demands made upon the hospital's capital by the necessity for providing modern up-to-date apparatus to utilise the more recent methods of treating the sick.

### The Cost of Special Departments.

In every department of hospital life this progressive advance in methods of treatment has to be chronicled, but the more striking advances are certainly to be found in what are known as the "special departments"—that is to say, in those branches of the institution which are devoted to certain special, as distinct from general, diseases. In the general ward, for example, all sorts of surgical or medical cases may be treated. Necessarily, recent advances in scientific method have made certain improvements, some of them very costly, indispensable in such a general ward if the work is to be carried on properly. But it is in the special department that the startling discrepancy between what sufficed twenty years ago and what is deemed indispensable to-day is most obvious, for it is here perhaps more than elsewhere that the elaboration of new apparatus and the improvement of old methods have been carried out in perfection.

As a type we may take the orthopædic department of a modern hospital. This is the department in which are treated cases of deformities which formerly were warded in the surgical beds and were left to the care of the general surgeon. The differentiation of orthopædics as a recognised specialty is a comparatively modern development; in most English hospitals, for instance, orthopædic cases are still treated by the general surgeon, and the orthopædic department, where it is recognised, exists only in name. But we are not now concerned with these conservative institutions, but with others which possess a definite, well-equipped orthopædic department, in charge of a well-trained specialist selected for his experience and his knowledge of the specialty with which he has to deal. The inception of such a department is due largely to the pioneering efforts of Professor Nicoladoni, of Graz, and Professor Hoffa, of Berlin, but their names are not singular, for they have been amply supported by orthopædists in France, Italy, and notably in Russia, where these special departments have been perfected to a degree which puts to shame many of our own orthopædic institutions. The modern conception of an orthopædic department is one fully equipped with special apparatus for the treatment of cases of deformity. As such it is seen at its best at an institution like the Rissoli at Bologna, or the Widener Home at Philadelphia, but as a special department attached to a hospital its best example is probably Professor Lange's new department at the general hospital at Munich.

The modern treatment of deformity may, broadly speaking, be divided into two main groups: treatment by operative procedure, and conservative treatment by means of massage, apparatus, and exercises. In both groups the advances have been striking. Twenty years ago a child suffering from congenital dislocation of the hip was doomed to undergo a prolonged, painful, and dangerous cutting operation which in eighty cases out of every hundred failed to relieve the deformity. Nowadays the treatment in such a case can be, and very often is, performed without admitting the patient into hospital. In the out-patient department the child is anæsthetised with ether, the dislocation is reduced by manipulative methods—the so-called bloodless method of Paci, Lorenz, or Schede—the limb put up in plaster-of-Paris bandages, and the child sent out, not to lie up for weeks but to walk about after a few days with a measure of ease and comfort which twenty years ago would have been deemed impossible of realisation. Three months later the plaster bandage is taken off, a new one substituted, and the child again allowed to walk; two other bandages are put on at intervals of three to six months, and after a year's treatment, most of which is carried on at home, the child is permitted to walk about without a splint, with the result that ultimately a perfect cure results, so that three years after the initial operation it would take an expert to say that the child's leg had ever been dislocated.

### Orthopædic Apparatus.

This, one of the most common procedures in the orthopædic department, needs special apparatus. To start with, there is the difficulty of reducing the dislocation. This operation can be carried out with perfect success by a skilled operator, who uses only his hands; where many patients are treated, however, it is found more convenient to have a special apparatus, such as that used by Lange or by Lovett, of Boston. The plaster-of-Paris bandages used must be of the best, and where an institution needs several hundreds of these, it finds it more economical to make than to buy them. For that purpose special bandage-rolling machines are required, and although the initial cost of installing such machinery, especially where driven by electricity, is high, they ultimately save the institution a good deal of money. Plaster bandages are used for a great many purposes in the orthopædic department. When a patient comes in with flat feet, the orthopædist takes a "mould" of his foot; from this mould a plaster

" cast " is in turn taken, which serves not only as a permanent record of the deformity but also as a type from which a corrected cast can be made, from which, again, the inset, of cork, metal, or celluloid, can be fashioned. Similarly, in a case of kyphosis, the surgeon takes a mould of the patient's bust; from this a cast is made, and on this is modelled the plaster jacket, this method resulting in a perfectly adapted jacket, which corrects the deformity or prevents it from progressing, as was the case so often with the old-style jackets fashioned on the patients themselves. In order to carry out all these methods, the department, to be complete, needs to have attached to it a properly fitted instrument-maker's cabinet. Here a skilled mechanician is installed, who does all the mechanical work under the surgeon's own supervision, not, as is again so often the case when instruments are ordered from outside makers, at his own discretion. The saving to the patients and the corresponding increase in value of the articles manufactured on the premises under the surgeon's own direction is easy to estimate, and when one compares the figures charged for splints, instruments, and appliances at a hospital which possesses its own mechanician with those ruling among instrument makers, the economy effected by the installation of a hospital mechanician is obvious.

A further use of apparatus in the orthopædic department is seen in the treatment of cases of fracture and kyphosis. In the latter, suspension apparatus is frequently necesary, either to enable the patient to be properly supported while a plaster jacket or corset is being put on or to keep him in a corrected position for a certain time as part of the curative treatment prescribed. The use of splints and supporting appliances is still very necessary, and here the hospital mechanician or instrument maker proves his value. He is on the spot and can be called in at once to fit the appliance to the surgeon's satisfaction; properly trained, he is able to make any splint which is used in modern times, so long as he has a model or scale drawing to go by. In the Continental and American clinics he has learned fully to appreciate the value of an adjunct with which English instrument makers and hospital workers do not seem to be acquainted, celluloid. As a material for splints and supports celluloid is almost ideal. It is elastic, light, clean, washable, lasting, and sanitary; its main objections are that it is not so cheap as plaster and that it is inflammable. The celluloid shavings are bought from the manufacturers in crates or boxes containing so many hundredweight; they are dissolved in acetone, which is bought from the manufacturing chemists in large carboys. The solution forms a stiff paste, which readily dries when exposed to the air and which serves admirably as a strengthening material for cork insets, or even as the basis for a plaster jacket, corset, or bed. This celluloid pot is one of the standbys of the Continental hospital-instrument maker; its use enables him to manufacture many appliances and supports with the least expenditure of time and trouble. As more stable materials he has " Durana " metal, brass, zinc, aluminium, or German silver or copper to work with—all materials which are readily moulded, and which can be adapted to suit particular cases with the greatest ease.

A case in point, which serves to show the usefulness of the celluloid pot, is furnished by a patient who comes up with a tuberculous knee. The proper treatment is to immobilise the joint, and to put on a splint which, while preventing the knee from being flexed, may be taken off with ease to allow inspection of the parts and the application of other methods of treatment, such as the Biers bandage, for instance. A plaster splint serves well, but it has the disadvantage that it is heavy, usually uncomfortable, and apt to get dirty. The surgeon therefore decides upon a celluloid splint. First of all, he takes a mould of the limb. The knee is cleaned, dusted with boracic powder, and a long fret-saw is laid longitudinally along it for the length that the permanent splint is to be. A well-plastered wet bandage is then quickly and lightly wound round the limb, and when this is dry the fret saw is made to cut through it, and the mould so formed is gently removed and allowed to dry. When dry it is wound round with a bandage, its lower end stopped up with paper, or the mould simply placed in a shallow dish, and it is filled with liquid plaster. When this is set the mould is removed, and the solid cast of the limb is now ready for the shaping of the celluloid mould which is to form the splint. A layer of Bavarian flannel is sewn round it, after the manner of a Croft, and this material is then painted with several coats, according to the strength that the surgeon desires the splint to be, of the celluloid paste. When these coats are dry, a further series of strengthening coats are applied, and finally the splint is taken off the cast, its edges trimmed, lined with chamois leather or felt, lacing holes punched in it, a tightening lace inserted, and it is ready for the patient, the final result being a perfectly reliable, light, easily washable, and therefore clean splint, which can be readily removed when required.

When we come to the operative procedures we find that the greatest advance in the treatment of deformities has been made in the direction of nerve surgery. Formerly, a child with a flail joint, paralysed by anterior poliomyelitis, had only two alternatives: one, the wearing of a permanent instrument, unwieldy, heavy, and cumbersome; the other, the amputation of a useless and inconvenient member. A third alternative, arthodesis, or the fixing of the joints of the limb so that a stiff limb resulted, remained, but the results obtained by this method of treatment were not so successful as to justify its general adoption. Now, thanks to the pioneering work of Professor Spitzy, there is a fourth alternative—the re-innervation of the paralysed limb by joining the " dead " nerves to still living nerves above the site of paralysis. The technique of this operation needs no description here; it is sufficient to say that the child is anæsthetised, the dead nerve exposed by an incision, the living nerve found, and the end of the dead one implanted into the living by an operation known as nerve grafting, the site of the anastomosis being covered with a delicate protective membrane made of scraped dog's artery (Foramitti's method). The results obtained, in selected cases, have been very striking, especially in Dr. Spitzy's hands, and the method has now been adopted by many other orthopædists. Similarly, cases of spasticity have been treated with success by nerve suture, or by division of the posterior nerve-roots, or by "nerve switching" operations. In minor degrees of paralysis of a limb alternative methods in use are tendon transplantation, by means of which the function of the paralysed joint is restored by making new traction points for alternative muscles to pull upon, or even by the formation of artificial tendons made of silk or cotton thread.

The after-treatment of all these cases of deformity needs great patience and often the employment of special apparatus, sometimes of a very expensive kind. The orthopædic department, therefore, must have a proper massage-room, in charge of a number of skilled and specially trained masseurs. Some enthusiasts even hold that it is necessary that the department should possess a large swimming-bath, but this is a counsel of perfection which few hospitals can follow!

On the other hand, it is indispensable that there should be a proper exercise-room, equipped with Swedish gymnastic and, more important even, with Zander apparatus—that is, with machines specially constructed so as to permit the patients to perform certain movements which exercise a particular group of muscles, while the antagonising groups of muscles are at rest. Such an installation can be seen at the Royal National Orthopædic Hospital in Great Portland Street, where it has proved of the greatest use in the after-treatment of cases. In cases of lateral curvature—a deformity which the orthopædist is frequently called upon to treat—the new methods of treatment by means of "crawling exercises," recommended by Professor Klapp and now so generally used in many clinics, demand a comparatively large-sized room, with a parquet or smooth oaken floor, or, at least, a large covered balcony, where the children can carry out the exercises under proper supervision. This supervision entails the training of special nurses. At Professor Klapp's clinic, for instance, a staff of ten nurses is told off to superintend the exercises performed by squads of children who come regularly every afternoon at two o'clock from the Berlin schools and undergo the treatment, which is combined with massage and manipulations, at the Royal Surgical Clinic in the Ziegelstrasse.

